# K-RET: knowledgeable biomedical relation extraction system

**DOI:** 10.1093/bioinformatics/btad174

**Published:** 2023-04-05

**Authors:** Diana F Sousa, Francisco M Couto

**Affiliations:** Departamento de Informática, Faculdade de Ciências, Universidade de Lisboa, Lisboa 1749-016, Portugal; Departamento de Informática, Faculdade de Ciências, Universidade de Lisboa, Lisboa 1749-016, Portugal

## Abstract

**Motivation:**

Relation extraction (RE) is a crucial process to deal with the amount of text published daily, e.g. to find missing associations in a database. RE is a text mining task for which the state-of-the-art approaches use bidirectional encoders, namely, BERT. However, state-of-the-art performance may be limited by the lack of efficient external knowledge injection approaches, with a larger impact in the biomedical area given the widespread usage and high quality of biomedical ontologies. This knowledge can propel these systems forward by aiding them in predicting more explainable biomedical associations. With this in mind, we developed K-RET, a novel, knowledgeable biomedical RE system that, for the first time, injects knowledge by handling different types of associations, multiple sources and where to apply it, and multi-token entities.

**Results:**

We tested K-RET on three independent and open-access corpora (DDI, BC5CDR, and PGR) using four biomedical ontologies handling different entities. K-RET improved state-of-the-art results by 2.68% on average, with the DDI Corpus yielding the most significant boost in performance, from 79.30% to 87.19% in F-measure, representing a *P*-value of $2.91\times 10^{-12}$.

**Availability and implementation:**

https://github.com/lasigeBioTM/K-RET.

## 1 Introduction

With the exponential increase in the number of research articles published in the last decades, researchers find it hard to keep up with all relevant information for their respective fields. The overwhelming number of research papers, particularly in the biomedical field, frequently makes it impossible for researchers and clinicians to be aware of all established entity associations and dissociations. This unawareness often leads to experimental repetition to prove or disprove hypotheses already studied. Further, even if the hypothesis is unique or new, the same inference could often be retrieved from knowledge about experiments done on similar entities. Automated biomedical relation extraction (RE) is a fundamental step toward aiding these researchers and clinicians in saving time and focusing their work on genuinely novel associations.

Through the years, several approaches have been employed to tackle biomedical RE, from straightforward rule-based approaches (Rinaldi et al. 2007, Kilicoglu et al. 2020) to entire machine learning dedicated systems (Abdelkader et al. 2021, Houssein et al. 2021), in which deep learning played a significant role (Dash et al. 2020). Successively, these approaches became more specialized in targeting multiple types of biomedical associations from protein–protein relations (Kim et al. 2006) to human phenotype-gene causality (Song et al. 2019, Sousa and Couto 2022). BERT (Kenton and Toutanova 2019) and their domain derivatives, such as BioBERT (Lee et al. 2020), SciBERT (Beltagy et al. 2019), and PubMedBERT (Gu et al. 2022) are the current state-of-the-art employed solutions. They not only successfully extract multiple types of relations from the text but can also, using their pretrained models, be easily integrated into newer systems targeting different Natural Language Processing tasks, such as Named-Entity Recognition (Nasar et al. 2022), Named-Entity Linking or Normalization (Ruas and Couto 2022), and Question Answering (Do and Phan 2022).

However, there are some caveats for most biomedical RE systems. The first limitation is their focus and specialization on one specific type of association (Hu et al. 2021), with some notable exceptions that are flexible to target more than one type (Song et al. 2019, Sousa and Couto 2020, 2022). The second limitation is that systems’ results frequently lack an explanation. One can not easily understand how a relation was found and what inferences a particular model made to reach an output. Finally, in close connection with making systems explainable, the third more relevant limitation is their general disregard for the vast repertoire of biomedical dedicated knowledge bases, particularly in the form of ontologies. This multitude of organized biomedical knowledge is freely available, but most systems still rely only on the information from the training data. Some exemptions recently made some strides into using knowledge in the general domain (Liu et al. 2020) and the biomedical/clinical domain (Hao et al. 2020). However, these are still limited in the domain’s specificity and inflexible to adding different or more structured knowledge.

Some of the most notary examples of organized biomedical knowledge are the Gene Ontology (GO; Ashburner et al. 2000, The Gene Ontology Consortium 2019), the Human Phenotype Ontology (HPO; Köhler et al. 2021), the Human Disease Ontology (DO; Schriml et al. 2022), the Chemical Entities of Biological Interest (ChEBI; Degtyarenko et al. 2008), and the Unified Medical Language System (UMLS; Bodenreider 2004). Respectively, these organized resources described connections between gene function descriptors, human phenotypes, human diseases, chemicals of biological interest, and clinical entities.

The work of Liu et al. (2020) is a recent and successful attempt for knowledge incorporation into multiple open and specific-domain tasks. Their K-BERT system was able to incorporate domain knowledge without creating a heterogeneous embedding space. The novelty in their approach is the creation of a knowledge layer that injects knowledge directly into the sentence. Then, they perform sentence tree re-arrangement before the embedding layer, with the addition of a soft-position embedding and a visible matrix to target possible readability problems from the re-arrangement. Also, it does not require pretraining of the BERT models it supports, making it suitable for users with limited computational resources (Zhao et al. 2019). However, their system has some major constrictions. First, the authors do not apply their approach to the RE task for the open or biomedical-specific domains. Second, the knowledge injection can only be used for single tokens within the sentence tree, making it miss knowledge associated with multi-token entities that are most prevalent in the biomedical domain. Third, the application of their system only allows for the injection of knowledge from one knowledge source, constraining data that intersects one or more domains (e.g. patient case reports that mention both the procedures done and the phenotypes associated with the patients). Finally, there is no possibility of injecting target knowledge into the entities of interest; one must always apply knowledge to all possible sentence tokens.

In this work, we designed a new approach to knowledge injection that is able to address the challenges stated in the last paragraph to create K-RET, a knowledgeable biomedical RE BERT-based system. K-RET is a flexible biomedical RE system, allowing for the use of any pretrained BERT-based system (e.g. SciBERT and BioBERT) to inject knowledge in the form of knowledge bases from a single source or multiple sources simultaneously. This knowledge can be applied to various contextualizing tokens or just to the tokens of the candidate relation for single and multi-token entities.

K-RET effectively improves the performance of baseline biomedical BERT-based models by an average of 2.68% on all datasets (DDI; Herrero-Zazo et al. 2013, Segura-Bedmar et al. 2014), BC5CDR (Li et al. 2016), and PGR-crowd (Sousa et al. 2019, 2020; Corpora). The most successful configuration is applying contextualized knowledge by adding two knowledge base entities per possible domain entity within each sentence tree (i.e. DDI Corpus plus ChEBI). This work resulted in the following main contributions:

- K-RET, a knowledgeable biomedical RE system that allows for the integration of any pre-trained BERT-based system.
- Approach to flexible injection of knowledge with multi-options:
  1. association of knowledge to single or multi-token entities in the sentence tree;
  2. add more than one knowledge source to inject knowledge of different domains;
  3. injection of knowledge into targeted or multiple contextualizing tokens in a sentence tree.

In Section 2, we describe K-RET, formally presenting the main architectural features. In Section 3, we describe the resources used (datasets and knowledge bases), parameters, training details, and the main results of different system configurations. These configurations are the baseline (i.e. without knowledge) and the use of target versus contextualized knowledge within three different biomedical RE datasets. Sections 4 and 5 discuss the previous results with targeted ablation studies and present the main conclusions and future work.

## 2 System and methods

**Figure 1.**
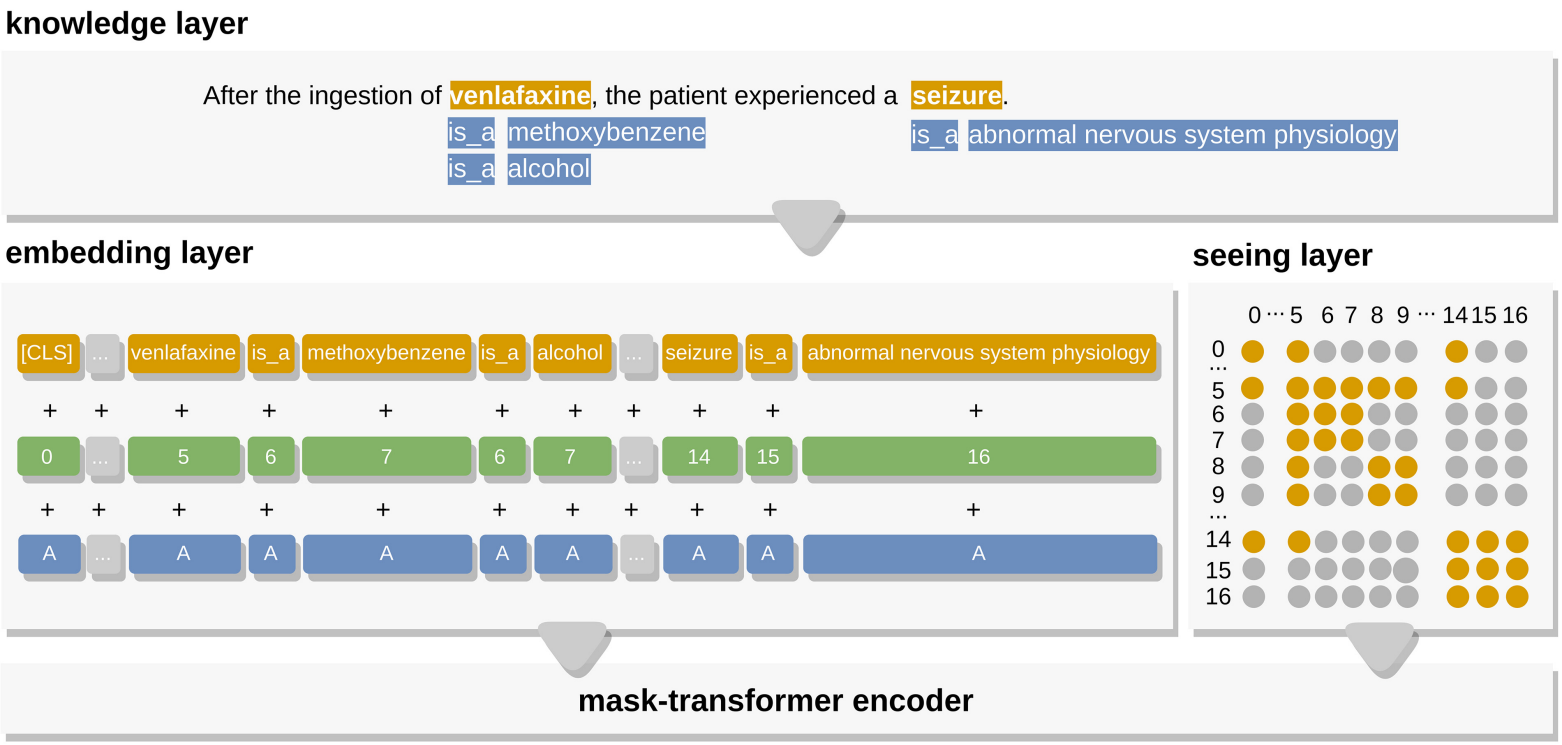
The structured layer representation of K-RET. In the pipeline, we retrieved a sentence from a biomedical relation extraction dataset with delimited target entities: venlafaxine and seizure. We added a Knowledge layer to these entities from associations made with their respective domain ontologies, the ChEBI and the Human DO. In the Embedding layer, the tokens are flattened into a sequence for token embedding. Then, the soft-position embedding is used along with the token embedding, and the tokens are tagged with A for segment embedding. The orange circles correspond to visible tokens in the Seeing layer, whereas the gray circles correspond to invisible ones. For instance, in row five venlafaxine (5) is visible to all tokens except the last two: is_a (15) and abnormal nervous system physiology (16). Finally, both layers, Embedding and Seeing, are fed to the Mask-transformer, corresponding to a stack of multiple mask-self-attention blocks. This last layer is masked to prevent the transformer from receiving structural information of the sentence tree. The sentence was simplified for readability purposes

In this section, we will present our system K-RET, represented in Fig. 1. We will describe each new system component and how they differ from the implementations from both BERT (Kenton and Toutanova 2019) and K-BERT (Liu et al. 2020), which was built using the UER platform (Zhao et al. 2019), starting by defining the general Notation and then detailing our additions in Architecture.

### 2.1 Notation

We define a sentence *s* as a sequence of tokens $t_{i}$, with length *n*:


(1) $$s=\{t_{0},t_{1},t_{2},\ldots ,t_{n}\}$$

Each token can represent one or more words that are included in the vocabulary *V*, $t_{i}\in V$. The Knowledge Base, *K*, consists of a collection of triples, *e*: where $t_{i}$ and $t_{k}$ are the descriptors of the entities, $r_{j}$ the relation between them, $r_{j}\in V$, and e∈K.


(2) $$e=(t_{i},r_{j},t_{k})$$

### 2.2 Architecture

The model architecture of K-RET is divided into four modules. The first module, a Knowledge layer, injects knowledge from the knowledge base in triples, expanding the original sentence into a knowledgeable sentence tree. The sentence tree is fed into simultaneously the Embedding layer (second module) and the Seeing layer (third module). Then, it is converted to token-level embedding representation and a visible matrix, as presented in Fig. 1. This matrix acts as a control to prevent the injected knowledge from altering the meaning of the original sentence with excessive knowledge. The embedding representation can then be fed into the Mask-transformer (fourth module).

K-BERT created a knowledge layer that injects knowledge and performs sentence tree conversion. On the other hand, our new knowledge layer was designed to address the three previously mentioned challenges. We detail how we tackled those limitations in the next sections: Multi-token entities, Multiple knowledge bases, and Contextual and targeted knowledge. Beyond the knowledge layer, we made changes to the original predictive pipeline by allowing the addition of class weights, a new type of data format, and the inclusion of any BERT-based pretrained model using the UER framework.

#### 2.2.1 Multi-token entities

For multi-token entities, we first considered all combinations of single tokens to generate all possible multi-tokens in the same input sentence. Thus, if a sentence has a number of tokens of 10, the number of combinations possible would be 55. This number can vary if the sentence has punctuation or other special characters. Then, through a lookup table, we can add knowledge to all combinations we can match in the chosen knowledge base, keeping the longest combinations of tokens (with increased specificity) when there is overlap. Finally, we reconstruct the sentence tree through a sliding window that goes through all the combinations with and without knowledge.

We introduced a multi-token entities option to address the limitation of only using one token, both in the sentence itself and in the knowledge to be injected. As it stands, the model did not allow associating knowledge to more than one token [e.g. ‘aralkylamino compound’ (original sentence tokens) *is_a* organic amino compound (added knowledgeable token)] nor associate multiple word knowledge to one or more words in the original sentence [e.g., dopamine (original sentence token) *is_a* ‘aralkylamino compound**’** (added knowledgeable tokens)].

#### 2.2.2 Multiple knowledge bases

To accommodate multiple knowledge bases, we expanded the number of lookup tables mentioned in the previous section. Thus, if we use more than one knowledge base, K-RET will look at the sentence a number of times corresponding to the number of knowledge bases. If there is complete or partial overlap between two or more competing knowledgeable tokens associated with an entity sentence and the number of association tokens surpasses the number of maximum tokens defined at the start, we keep the ones with the highest information content.

The multiple knowledge bases option resolves the limitation of using only one knowledge base at a time. Therefore if we have, as in the biomedical domain, knowledge bases targeting different types of entities, we have to choose which one we want to use to inject knowledge into the original sentence.

#### 2.2.3 Contextual and targeted knowledge

Finally, we decided to add the possibility of only injecting knowledge into the entities in consideration for relation assessment, maintaining the native option of adding knowledge to all entities present in the sentence.

To define the targeted entities, we used the tags < e > and </e > to delimit the entities in the candidate relation. The tags allowed injecting knowledge directly into the candidate entities. K-RET ignores those tags when using contextual knowledge for knowledge injection, adding knowledge to entities as described previously.

Hence, as a result of the three additions, given an input sentence $s=\{t_{0},t_{1},t_{2},\ldots ,t_{n}\}$ and one or more knowledge bases, our knowledge layer outputs a sentence tree: which results from (i) querying all entity names involved in the sentence *s*, independently from their length and selecting correspondent triples from *K*, and (ii) adding the triples to their correspondent position. Figure 1 illustrates the structure of the sentence tree *st* and an example retrieved from our data. While the sentence tree can have multiple branches, the depth is fixed to 1, not deriving branches iteratively to better preserve the original sentence meaning.


(3) $$st=\{t_{0},t_{1},t_{2},\ldots ,t_{i}\{(r_{i0},t_{i0}),\ldots ,(r_{ik},t_{ik})\},\ldots ,t_{n}\}$$

## 3 Implementation

In this article, we used three openly available RE datasets to train K-RET: DDI Corpus (Herrero-Zazo et al. 2013, Segura-Bedmar et al. 2014; drug–drug interactions), PGR-crowd Corpus (Sousa et al. 2019, 2020; human phenotype–gene interactions), and BC5CDR Corpus (Li et al. 2016; chemical–disease associations). Along with the written information in these RE datasets, we used four knowledge bases related to the four types of entities identified within those datasets to add extra entity information to the K-RET system. These knowledge bases are HPO (Köhler et al. 2021), DO (Schriml et al. 2022), CHEBI (Degtyarenko et al. 2008), and GO (Ashburner et al. 2000, The Gene Ontology Consortium 2019). In this section, we present the different experiments made to access our system using the aforementioned datasets and knowledge bases, with parameters and training details tuned for the specificities of each dataset. We also explore and present the results for the usage of full knowledge or just entity knowledge, as described in the previous section.

### 3.1 Datasets

**Figure 2.**
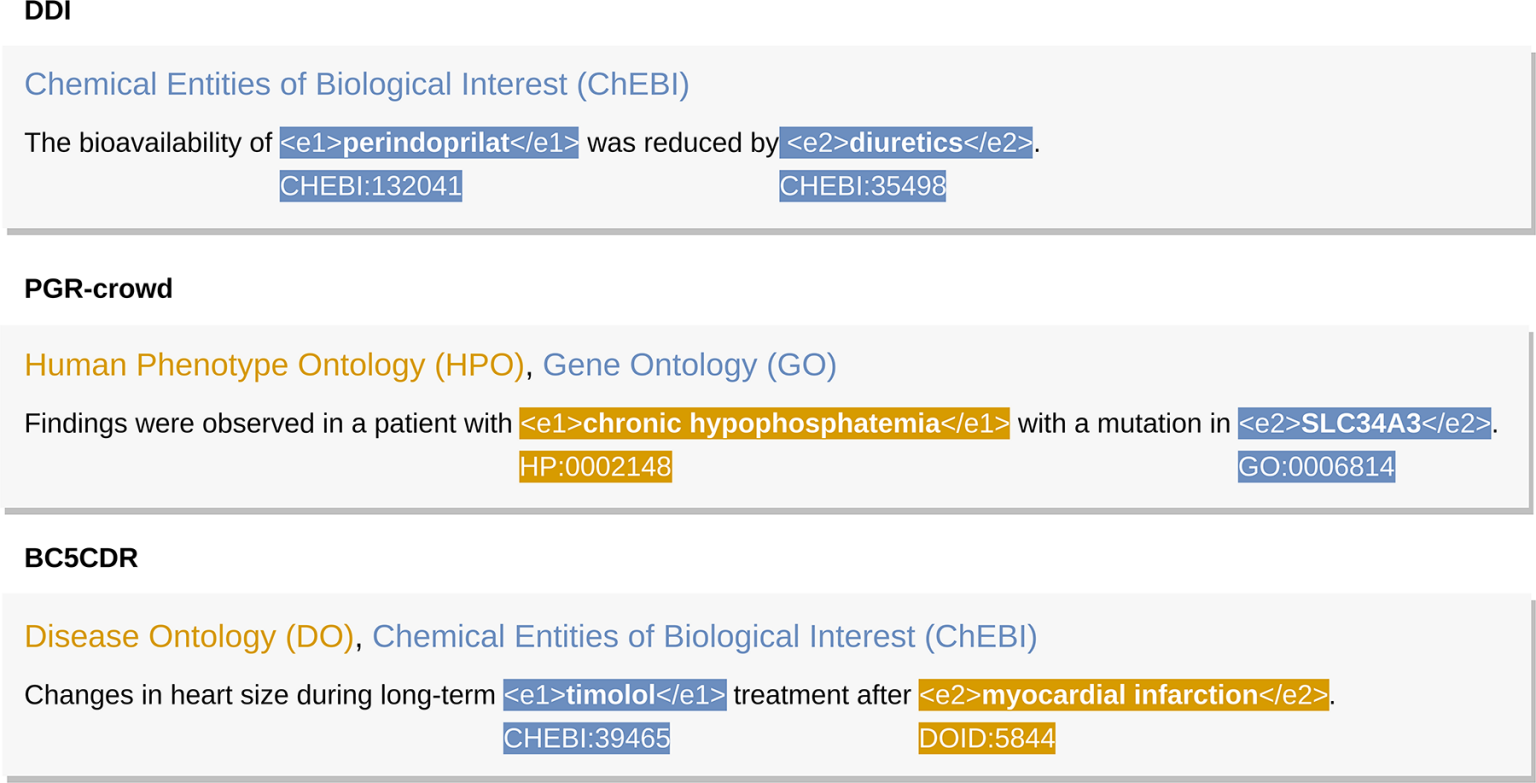
Three sentence examples for each dataset, DDI Corpus, PGR-crowd Corpus, and BC5CDR Corpus. The entities in each candidate relation are linked to corresponding knowledge bases

Table 1 represents the relations types and counts for each dataset. The *no_relation* label accounts for entities present in the same sentence but that do not share a relation. The commonly used DDI Corpus presents four types of relations: *effect* to describe an effect or pharmacodynamics mechanism, *mechanism* to describe a pharmacokinetic mechanism, *advice* to describe semantic relations between drugs regarding recommendation, and *int* for relations where there is no further information. Both the PGR-crowd and BC5CDR Corpora are binary in terms of relation classification. These two datasets only classify if the relation is present (*true*) or not (*false*). Figure 2 presents an example sentence for each dataset.

**Table 1.** The main statistics of DDI Corpus, PGR-crowd Corpus, and BC5CDR Corpus regarding the RE task.

Dataset	Relation type				
	True				No-relation/False
	Effect	Advice	Mechanism	Int	
DDI	2026	1616	1047	278	29 245
PGR-crowd	5498				626
BC5CDR	1448				2294

We applied class weights to all datasets to normalize the different types of relations and distributions. Additionally, to train and evaluate all models on the same cross-validation splits, we split each dataset into training (60%), validation (10%), and test (30%) sets. Also, our metrics presented below are displayed considering the weighted average for each type of relation to further account for the datasets’ imbalances.

### 3.2 Knowledge bases

Several knowledge bases target biomedical entities, expanding on our information about them and their inherent relationships. Some of the knowledge bases or ontologies we can link to our target entities were mentioned above and are characterized in Table 2.

**Table 2.** The main characteristics of the following knowledge bases: HPO, DO, ChEBI, and GO. All types of relations are transitive.^a^

Knowledge bases	Number of concepts	Type of relations	Specific characteristics
HPO	15 670	*is-a*	Five sub-ontologies, from which *phenotypic abnormality* is the most prevalent
DO	13 355	*is-a*	Human-specific
ChEBI	153 795	*is-a*	Refers to small molecular entities
		*has-part*	
		*is-conjugate-base-of*	
		*is-conjugate-acid-of*	
		*is-tautomer-of*	
		*is-enantiomer-of*	
		*has-functional-parent*	
		*has-parent-hydride*	
		*is-substituent-group-from*	
		*has-role*	
GO	43 613	*is-a*	Three subontologies, molecular function, biological process, and cellular component
		*part-of*	
		*has-part*	
		*regulates*	
		*negatively-regulates*	
		*positively-regulates*	

a All knowledge bases were consulted on 20 April 2022.

With few code adjustments, one can easily integrate more knowledge into K-RET in the form of other knowledge bases and has the flexibility to test different combinations swiftly.

### 3.3 Parameters and training details

For each dataset, we empirically determined the best set of parameters. K-RET used a batch size of 32 for all datasets. The number of epochs ranged from 30 for DDI Corpus to 20 for PGR-crowd and BC5CDR Corpora (due to differences in dataset size). All other parameter settings were maintained from the original BERT model. The added knowledge only played a role in the fine-tuning and prediction stage.

**Figure 3.**
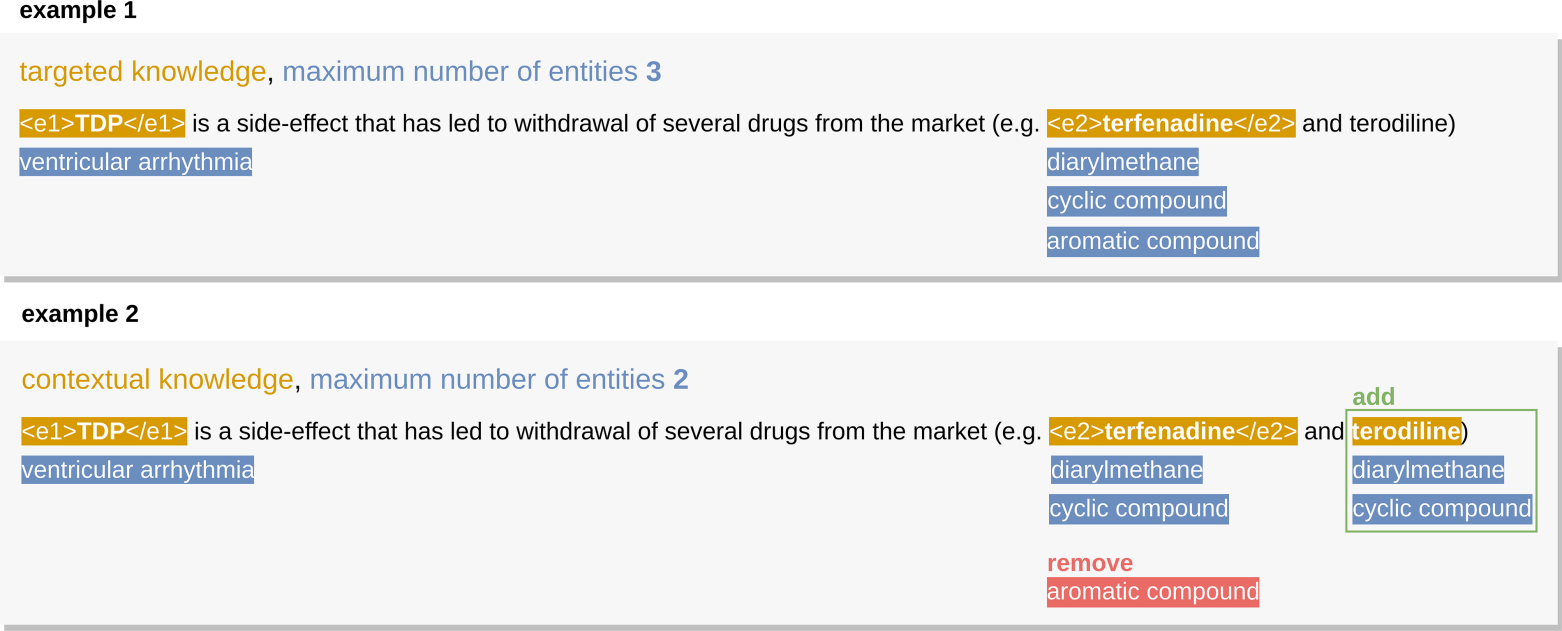
Novelties of K-RET’s knowledge injection layer. Example 1 presents a modality where we only add knowledge to entities in the candidate relation (targeted knowledge) and limit the number of knowledge base entities assigned to each entity (maximum number of Entities 3). In Example 2, we present the same sentence with the option of adding knowledge to all entities regardless if they are in the candidate relation (contextual knowledge) and limit the number of knowledge base entities assigned to each entity to two (maximum number of Entities 2). From Examples 1 to 2, we add a third knowledgeable entity (terodiline) and remove one of the knowledge base entities added (aromatic compound). The first entity can only be linked to one knowledge entity because (ventricular arrhythmia) no other association is established in the knowledge base

For the added knowledge, we varied the number of knowledge base entities allowed to link to each dataset entity in a candidate relation (or not). This variation ranged from two to five knowledge base entities per dataset entity. Our baseline represents the performance of the BERT-based systems without any added knowledge. When we refer to targeted knowledge, we mean just adding knowledge to entities in a candidate relation versus contextual knowledge, where we can add knowledge to all entities in the dataset. Figure 3 further elucidates the differences in the addition of knowledge. For the DDI Corpus, we used the ChEBI ontology; for the PGR-, we used the HPO and the GO ontologies; and for the BC5CDR Corpus, we used the DO and ChEBI ontologies, as represented in Fig. 2.

All models were trained on three Tesla M10 GPUs, taking, on average for each model, 24 h (DDI Corpus), 1 h (PGR-crowd Corpus), and 2 h (BC5CDR Corpus). Each result presented in the following sections represents the averaged metrics for three runs except when it explicitly says otherwise, and each metric represents the weighted-averaged of the different labels.

### 3.4 Results

As mentioned in the previous sections, we divided our K-RET experiments into baseline, where we ran the BERT-based models without additional knowledge, targeted knowledge added to the entities in the candidate relation, and contextual knowledge added to all relevant entities in the sentence. We considered an entity relevant if present in the chosen knowledge source (i.e., a part of the domain knowledge considered).

#### 3.4.1 Baseline

To choose the best-performing BERT-based biomedical model for each of the three datasets, we used four of the most widely used systems: BERT (Kenton and Toutanova 2019), BioBERT (Lee et al. 2020), SciBERT (Beltagy et al. 2019), and PubMedBERT (Gu et al. 2022) integrated into K-BERT (Liu et al. 2020) modified to perform RE.

Table 3 reports the main results of testing the three datasets over the different BERT-based systems. For all three datasets, SciBERT is the best-performing system. Therefore, in our following experiments, we used SciBERT as the baseline system to which we added a knowledge layer.

**Table 3.** The baseline performance of the four models for each dataset.^a^

Dataset	Model	Precision	Recall	F-measure	Accuracy
PGR-crowd	BERT	0.7247	0.7766	0.7332	0.7765
	SciBERT	**0.7680**	**0.8002**	**0.7462**	**0.7999**
	BioBERT	0.6224	0.7888	0.6957	0.7888
	PubMedBERT	0.7201	0.7834	0.7191	0.7833
DDI	BERT	0.7764	0.7798	0.7775	0.7796
	SciBERT	**0.7906**	**0.7964**	**0.7930**	**0.7960**
	BioBERT	0.7796	0.7648	0.7680	0.7647
	PubMedBERT	0.7801	0.7227	0.7473	0.7227
BC5CDR	BERT	0.5804	0.5614	0.5670	0.5615
	SciBERT	**0.6266**	**0.6364**	**0.6289**	**0.6363**
	BioBERT	0.6150	0.6132	0.6142	0.6131
	PubMedBERT	0.6091	0.6218	0.6113	0.6217

a The specific models used were bert-base-uncased (BERT), scibert_scivocab_uncased (SciBERT), biobert-base-cased-v1.2 (BioBERT), and BiomedNLP-PubMedBERT-base-uncased-abstract-fulltext (PubMedBERT).

The highest scores for each metric are presented in bold.

#### 3.4.2 Targeted knowledge

**Figure 4.**
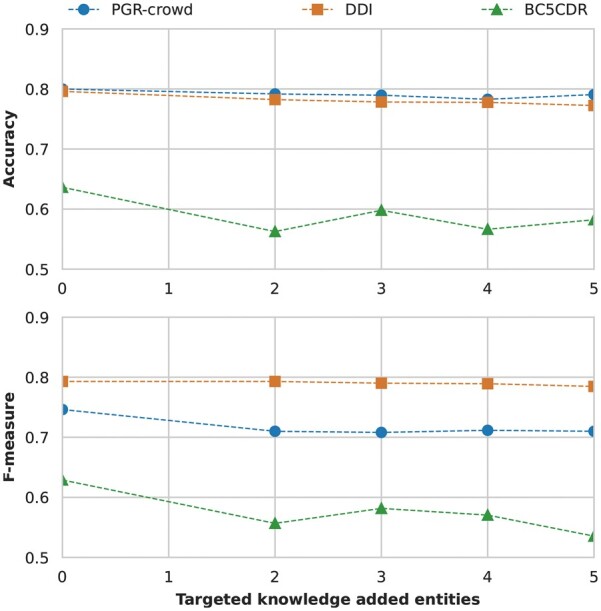
The performance of the targeted knowledge K-RET configuration for the three datasets in Accuracy and F-Measure (top and bottom graphs, respectively) regarding the different number of added knowledge base entities

For targeted knowledge, we added from two to five knowledge base entities to each entity in the candidate relation, as shown previously in Example 1 (Fig. 3). Although we define the number of knowledge base entities to add, we are always limited by how many entities each entity is linked to in the knowledge base itself. Figure 4 presents the performance of the three datasets from no added knowledge (baseline) to five added entities per entity in the candidate relation.

Figure 4 shows that none of the K-RET models trained performs better than the baseline at any number of knowledge base-added entities. For all datasets, there is a slight decrease in performance, with the BC5CDR Corpus having a small increase in performance when the number of added knowledge base entities equals three.

#### 3.4.3 Contextual knowledge

**Figure 5.**
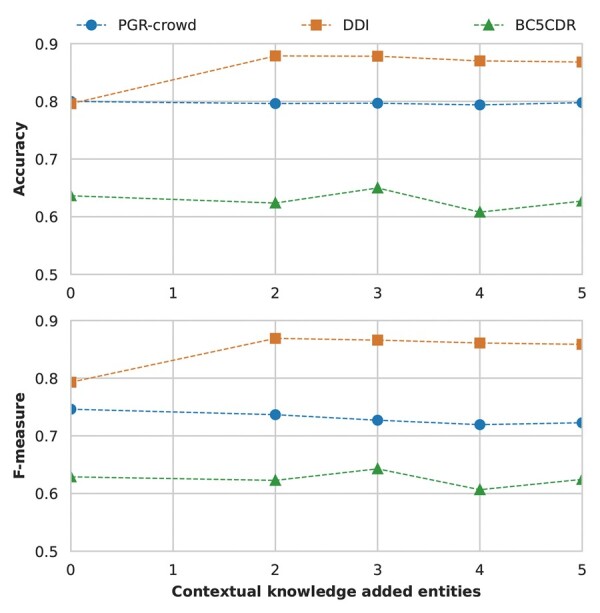
The performance of the contextual knowledge K-RET configuration for the three datasets n Accuracy and F-Measure (top and bottom graphs, respectively) regarding the different number of added knowledge base entities

We followed the same configuration mentioned above for contextual knowledge from two to five knowledge base entities added to each relevant entity in the sentence. Figure 5 presents the performance of the three datasets from no added knowledge (baseline) to five added entities per relevant entity.

Figure 5 shows a performance increase from baseline to K-RET on the DDI and BC5CDR Corpora. For the PGR-crowd Corpus, while the performance is better than for targeted knowledge, the baseline performance still outperforms K-RET. Similarly, the BC5CDR’s best performance is when the number of added knowledge base entities equals three, but this time surpasses the baseline.

Table 4 presents the best results for each main model configuration (targeted and contextual), including the contextual knowledge configuration over 10 runs to determine the statistical significance of the best configuration accurately and the corresponding baseline considered previously and regarding the majority label. The baseline${}_{\text{ML}}$ represents the results if we assign the same label (majority label) to all the test relations.

**Table 4.** The final K-RET performance results.^a^

Dataset	Model	Precision	Recall	F-measure	Accuracy
PGR-crowd	Baseline${}_{\text{ML}}$	0.6222	0.7888	0.6957	0.7888
	Baseline	**0.7680**	**0.8002**	**0.7462**	**0.7999**
	TK-RET	0.7891	0.7914	0.7100	0.7917
	CK-RET	0.7614	0.7960	0.7367	0.7962
	CK-RET_10_	0.7441	0.7933	0.7206	0.7934
DDI	Baseline${}_{ML}$	0.7267	0.8525	0.7846	0.8525
	Baseline	0.7906	0.7964	0.7930	0.7960
	TK-RET	0.8132	0.7820	0.7930	0.7823
	CK-RET	0.8674	0.8791	0.8690	0.8788
	CK-RET_10_	**0.8704**	**0.8805**	**0.8719**	**0.8804**
BC5CDR	Baseline${}_{ML}$	0.3803	0.6167	0.4705	0.6167
	Baseline	0.6266	0.6364	0.6289	0.6363
	TK-RET	0.5838	0.5977	0.5816	0.5980
	CK-RET	**0.6412**	**0.6499**	**0.6428**	**0.6498**
	CK-RET_10_	0.6317	0.6348	0.6309	0.6347

a The best-performing models for each dataset concerning baseline considering the majority label, baseline, targeted knowledge, contextual knowledge, and contextual knowledge over ten runs. To facilitate table readability, we omitted the standard deviation values that range from 0.005 to 0.029 across all datasets due to the low impact these have on the interpretation of the final results.

The highest scores for each metric are presented in bold.

The results show that the contextual knowledge configuration over three runs prevails as the best model in two datasets (DDI and BC5CDR), with the targeted knowledge configuration not surpassing the baseline for any dataset. However, the BC5CDR Corpus presents a p-value of 0.8108 for F-measure and 0.8681 for accuracy (*α* = .05), demonstrating a lack of significant difference between the means of Baseline and CK-RET_10_. Nonetheless, the DDI Corpus presents a p-value of $2.91\times 10^{-12}$ for F-measure and $1.44\times 10^{-12}$ for accuracy (*α* = .05), validating a significant difference between the means of Baseline and CK-RET_10_. The *P*-values were determined using a one-tailed *t*-test.

Table 5 reflects the specific performance of the DDI Corpus per type of relation (*effect*, *advice*, *mechanism*, *int*, and *false*) for each main model configuration. Supplementary Tables SA1 and SA2 present the same results for PGR-crowd and BC5CDR Corpora. For the DDI Corpus, we also performed ablation regarding multi-token entities by limiting the association of knowledge to the first entity within the multi-token considered. We obtained an F-measure of 0.8663 and an accuracy of 0.8732.

**Table 5.** DDI Corpus performance per type of relation.

Metrics	Model	Type				
		Effect	Advice	Mechanism	Int	False
Precision	Baseline	0.1770	0.0890	0.2720	0.2800	0.8060
	TK-RET	0.2747	0.3053	0.3623	0.2360	0.9020
	CK-RET	0.5137	**0.6720**	**0.5757**	0.5963	0.9163
	CK-RET_10_	**0.5524**	0.6370	0.5411	**0.6659**	**0.9194**
Recall	Baseline	0.2000	0.0730	0.1810	0.3850	0.9040
	TK-RET	0.4677	0.3490	0.5127	0.0160	0.8487
	CK-RET	0.4533	0.2937	0.4950	**0.4340**	**0.9600**
	CK-RET_10_	**0.4758**	**0.3226**	**0.5318**	0.3946	0.9579
F-measure	Baseline	0.1880	0.0800	0.2170	0.3250	0.9000
	TK-RET	0.3423	0.3240	0.4187	0.0293	0.8743
	CK-RET	0.4810	0.4077	0.5317	**0.5017**	0.9377
	CK-RET_10_	**0.5099**	**0.4266**	**0.5329**	0.4924	**0.9384**

## 4 Discussion

From the experiences conducted throughout the previous section, we can infer that adding knowledge can significantly improve the state-of-the-art performance of one of the most used biomedical RE datasets, mainly when using contextual knowledge. However, there is a difference in how significantly we can improve performance, from nothing (PGR-crowd Corpus) to over 0.076 percentage points (DDI Corpus). Several factors can explain the differences in performance for the different datasets, such as dataset size and label distribution, knowledge base size and coverage of the dataset entities, or the average number of knowledge base entities that can be linked to each dataset entity.

For the PGR-crowd Corpus, while all entities are linked to one of two knowledge bases, the HPO or the GO, the linking to the GO is already second handed. In the PGR-crowd Corpus, the authors recognized gene entities and posteriorly linked them to their most representative GO term. We do not use their gene identifiers directly because these biomedical entities are not directly represented in any hierarchic knowledge base. Thus, we used the GO terms to add further information and, consequently, distanced ourselves from the original gene entity. However, what made the most significant impact in the lack of increase in performance is the distribution of labels being predominantly *true*. Identifying undetected *true* relations is more challenging than if the dataset was more balanced (e.g. the BC5CDR Corpus) or with the inverse distribution (e.g. the DDI Corpus). Also, for the specific case of PGR-crowd Corpus, there could be ambiguity if an acronym describes a human phenotype or a gene since these can often overlap. This ambiguity is an open problem, and in this work, we opted not to consider human phenotype entities acronyms for contextual knowledge, which could be made us lose valuable information.

With the BC5CDR Corpus, K-RET only surpassed the baseline when adding contextual knowledge by slightly over 1% in both F-measure and accuracy and was unsuccessful at demonstrating a significant difference between the baseline and the best-performing configuration. Although the impact on performance is not preeminent, the fact that this dataset is denser in the number of relevant entities per sentence and more evenly distributed than the PGR-crowd Corpus increases the impact of the addition of knowledge. This behavior occurs when knowledge is added directly to the candidate relation’s target entities and peripherally to other relevant entities.

The DDI Corpus is unbalanced in favor of *no_relation*/*false* labels. Therefore, finding *true* relations is more challenging. This dataset’s distribution of labels is ideal for increasing the impact of adding knowledge. The label distribution, the highly dense text in relevant entities, and the ampler size of the knowledge base used, ChEBI, all contribute to the rise in performance by adding contextual knowledge. The improvements from baseline were 7.60% for contextual knowledge in F-measure. We also verified that adding knowledge made a bigger impact on system performance than using multi-token entities by contributing to an average improvement of 0.0704 data points in F-measure and 0.0756 in accuracy.

K-RET was able to design a more efficient and flexible knowledge layer than the previous attempt in the work of Liu et al. (2020) by applying knowledge injection to the biomedical RE task. First, by proving the utility of contextualizing tokens, with substantial improvements in one of the three datasets described above from targeted to contextual token usage. Second is the possibility of adding more than one knowledge source to cover the domains of all relevant entities mentioned in the training data. Third, by incorporating the option of adding knowledge to multi-token entities instead of only single token. We determined experimentally for the DDI Corpus the multi-token approach to be more thorough, which can be explained by those entities constituting the majority of biomedical entities described in the datasets used for testing.

## 5 Conclusion

This article proposed a new biomedical RE system, K-RET, that incorporates knowledge in the form of ontologies into BERT-based systems to enrich and complement the data used for training. K-RET is a flexible system that can handle different associations, integrate knowledge from multiple sources, define where to apply the knowledge, and deal with multi-token entities. We used three independent and open-access RE datasets to test our system concerning different types of biomedical entities with different characteristics (i.e. label distribution). Allied with the three datasets, we used four knowledge bases to inject knowledge according to the type of entities involved in the candidate relations. The best-performing dataset was the DDI Corpus allied with the ChEBI knowledge base, with significant average improvements compared with the baseline in the order of 7.60% and 8.28% for F-measure and accuracy, respectively. For the BC5CDR Corpus, we also had modest improvements (an average of 1.39% and 1.35% for F-measure and accuracy), even though we did not find these results significant. Differently, for the PGR-crowd Corpus, there was no significant change in performance from the addition of knowledge. Thus, we concluded that the label distribution and relevant entity density within the training data significantly affect how our system performs. Nevertheless, we successfully demonstrated the power of adding external knowledge to training data and how to accommodate it to different domain data.

In a nutshell, the K-RET system successfully added contextual knowledge, more than one knowledge source, and knowledge to multi-token entities.

For future work, we would like to explore the impact of different knowledge bases and combinations within the ones we already used. Also, we would like to analyze further and examine the implications of peripherical contextual entities on assigning a label for a candidate relation and entities overlapping within different knowledge bases.

## Supplementary Material

btad174_Supplementary_Data

## Data Availability

The datasets and biomedical ontologies were derived from sources in the public domain: PGR-crowd Corpus: https://github.com/lasigeBioTM/PGR-crowd. DDI Corpus: https://github.com/isegura/DDICorpus. BC5CDR Corpus: https://github.com/isegura/DDICorpus. Human Phenotype Ontology (HPO): https://hpo.jax.org/app/data/ontology. Human Disease Ontology (DO): https://www.ebi.ac.uk/ols/ontologies/doid. Chemical Entities of Biological Interest (ChEBI): https://www.ebi.ac.uk/chebi/. Gene Ontology (GO): http://geneontology.org/.

## References

[btad174-B1] Abdelkader W , Navarro T, Parrish R et al Machine learning approaches to retrieve high-quality, clinically relevant evidence from the biomedical literature: systematic review. JMIR Med Inform 2021;9:e30401. 34499041 10.2196/30401 PMC8461527

[btad174-B2] Ashburner M , Ball CA, Blake JA et al Gene ontology: tool for the unification of biology. Nat Genet 2000;25:25–9. 10802651 10.1038/75556 PMC3037419

[btad174-B3] Beltagy I , Lo K, Cohan A. SciBERT: A pretrained language model for scientific text. In: *Proceedings of the 2019 Conference on Empirical Methods in Natural Language Processing and the 9th International Joint Conference on Natural Language Processing (EMNLP-IJCNLP)*, pp. 3615–20, Hong Kong, China: Association for Computational Linguistics, 2019.

[btad174-B4] Bodenreider O. The unified medical language system (UMLS): integrating biomedical terminology. Nucleic Acids Res 2004;32:D267–70. 14681409 10.1093/nar/gkh061 PMC308795

[btad174-B5] The Gene Ontology Consortium. The gene ontology resource: 20 years and still going strong. Nucleic Acids Res 2019;47:D330–8. 30395331 10.1093/nar/gky1055 PMC6323945

[btad174-B6] Dash S , Acharya BR, Mittal M et al 2020. Deep Learning Techniques for Biomedical and Health Informatics. Cham, Switzerland: Springer.

[btad174-B7] Degtyarenko K , De Matos P, Ennis M et al ChEBI: a database and ontology for chemical entities of biological interest. Nucleic Acids Res 2008;36:D344–50. 17932057 10.1093/nar/gkm791 PMC2238832

[btad174-B8] Do P , Phan TH. Developing a BERT based triple classification model using knowledge graph embedding for question answering system. Appl Intell 2022;52:636–51.

[btad174-B9] Gu Y , Tinn R, Cheng H et al Domain-specific language model pretraining for biomedical natural language processing. ACM Trans Comput Healthc 2022;3:1–23.

[btad174-B10] Hao B , Zhu H, Paschalidis IC. Enhancing clinical bert embedding using a biomedical knowledge base. In: *28th International Conference on Computational Linguistics (COLING 2020)*, 2020.

[btad174-B11] Herrero-Zazo M , Segura-Bedmar I, Martínez P et al The ddi corpus: an annotated corpus with pharmacological substances and drug–drug interactions. J Biomed Inform 2013;46:914–20. 23906817 10.1016/j.jbi.2013.07.011

[btad174-B12] Houssein EH , Mohamed RE, Ali AA. Machine learning techniques for biomedical natural language processing: a comprehensive review. IEEE Access 2021;9:140628–53.

[btad174-B13] Hu L , Wang X, Huang Y-A et al A survey on computational models for predicting protein–protein interactions. Brief Bioinform 2021;22:bbab036. 33693513 10.1093/bib/bbab036

[btad174-B14] Kenton JDM-WC , Toutanova LK. BERT: Pre-training of deep bidirectional transformers for language understanding. In: *Proceedings of NAACL-HLT*, pp. 4171–86, 2019.

[btad174-B15] Kilicoglu H , Rosemblat G, Fiszman M et al Broad-coverage biomedical relation extraction with semrep. BMC Bioinformatics 2020;21:1–28. 32410573 10.1186/s12859-020-3517-7 PMC7222583

[btad174-B16] Kim J-J , Zhang Z, Park JC et al Biocontrasts: extracting and exploiting protein–protein contrastive relations from biomedical literature. Bioinformatics 2006;22:597–605. 16368768 10.1093/bioinformatics/btk016

[btad174-B17] Köhler S , Gargano M, Matentzoglu N et al The human phenotype ontology in 2021. Nucleic Acids Res 2021;49:D1207–17. 33264411 10.1093/nar/gkaa1043 PMC7778952

[btad174-B18] Lee J , Yoon W, Kim S et al BioBERT: a pre-trained biomedical language representation model for biomedical text mining. Bioinformatics 2020;36:1234–40. 31501885 10.1093/bioinformatics/btz682 PMC7703786

[btad174-B19] Li J , Sun Y, Johnson RJ et al Biocreative v cdr task corpus: a resource for chemical disease relation extraction. Database 2016;2016:baw068. 27161011 10.1093/database/baw068 PMC4860626

[btad174-B20] Liu W , Zhou P, Zhao Z et al K-BERT: Enabling language representation with knowledge graph. In: *Proceedings of the AAAI Conference on Artificial Intelligence*, Vol. 34, pp. 2901–8, 2020.

[btad174-B21] Nasar Z , Jaffry SW, Malik MK. Named entity recognition and relation extraction: state-of-the-art. ACM Comput Surv 2022;54:1–39.

[btad174-B22] Rinaldi F , Schneider G, Kaljurand K et al Mining of relations between proteins over biomedical scientific literature using a deep-linguistic approach. Artif Intell Med 2007;39:127–36. 17052900 10.1016/j.artmed.2006.08.005

[btad174-B23] Ruas P , Couto FM. NILINKER: attention-based approach to NIL entity linking. J Biomed Inform 2022;132:104137. 35811025 10.1016/j.jbi.2022.104137

[btad174-B24] Schriml LM , Munro JB, Schor M et al The human disease ontology 2022 update. Nucleic Acids Res 2022;50:D1255–61. 34755882 10.1093/nar/gkab1063 PMC8728220

[btad174-B25] Segura-Bedmar I , Martínez P, Herrero-Zazo M. Lessons learnt from the ddiextraction-2013 shared task. J Biomed Inform 2014;51:152–64. 24858490 10.1016/j.jbi.2014.05.007

[btad174-B26] Song L , Zhang Y, Gildea D et al Leveraging dependency forest for neural medical relation extraction. In: *Proceedings of the 2019 Conference on Empirical Methods in Natural Language Processing and the 9th International Joint Conference on Natural Language Processing (EMNLP-IJCNLP)*, pp. 208–18, 2019.

[btad174-B27] Sousa D , Couto FM. BiOnt: deep learning using multiple biomedical ontologies for relation extraction. In: *European Conference on Information Retrieval*, pp. 367–74. Springer, 2020.

[btad174-B28] Sousa D , Couto FM. Biomedical relation extraction with knowledge graph-based recommendations. IEEE J Biomed Health Inform 2022;26:4207–17. 35536818 10.1109/JBHI.2022.3173558

[btad174-B29] Sousa D , Lamurias A, Couto FM. A silver standard corpus of human phenotype-gene relations. In: *Proceedings of the 2019 Conference of the North American Chapter of the Association for Computational Linguistics: Human Language Technologies*, Vol. 1 (Long and Short Papers), pp. 1487–92, 2019.

[btad174-B30] Sousa D , Lamurias A, Couto FM. A hybrid approach toward biomedical relation extraction training corpora: combining distant supervision with crowdsourcing. Database, pp. 1–15. 2020;2020. 10.1093/database/baaa104 PMC7706181 33258966

[btad174-B31] Zhao Z , Chen H, Zhang J et al UER: An open-source toolkit for pre-training models. In: *EMNLP-IJCNLP 2019*, p. 241, 2019.

