# Supplementary material for: K-RET: knowledgeable biomedical relation extraction system
Source: Bioinformatics. 2023 Apr 5;39(4):btad174. doi: 10.1093/bioinformatics/btad174 (PMC10112952; doi:10.1093/bioinformatics/btad174)
Supplement: btad174_Supplementary_Data [file btad174_supplementary_data.pdf]

Supplementary Data

Table A1 and Table A2 present the PGR-crowd and BC5CDR Corpora performances per type o relation.

Table A1. PGR-crowd performance per type of relation

Metrics	Model	Type	
		True	False
Precision	Baseline	**0.8090**	0.6150
	TK-RET	0.7920	**0.7720**
	CK-RET	0.8047	0.5997
	CK-RET_{10}	0.7980	0.5429
Recall	Baseline	0.9770	**0.1400**
	TK-RET	**0.9940**	0.0350
	CK-RET	0.9790	0.1123
	CK-RET_{10}	0.9883	0.0651
F-measure	Baseline	**0.8850**	**0.2280**
	TK-RET	0.8827	0.0653
	CK-RET	0.8833	0.1890
	CK-RET_{10}	0.8829	0.1144

Table A2. BC5CDR performance per type of relation

Metrics	Model	Type	
		True	False
Precision	Baseline	0.5310	0.6860
	TK-RET	0.4703	0.6543
	CK-RET	**0.5537**	**0.6957**
	CK-RET_{10}	0.5304	0.6947
Recall	Baseline	0.4440	0.7560
	TK-RET	0.3623	0.7440
	CK-RET	0.4610	**0.7673**
	CK-RET_{10}	**0.4862**	0.7272
F-measure	Baseline	0.4840	0.7190
	TK-RET	0.4007	0.6940
	CK-RET	0.5030	**0.7297**
	CK-RET_{10}	**0.5046**	0.7094
